# Development and validation of an APCI-MS/GC–MS approach for the classification and prediction of Cheddar cheese maturity

**DOI:** 10.1016/j.foodchem.2015.05.096

**Published:** 2016-01-01

**Authors:** Heng Hui Gan, Bingnan Yan, Robert S.T. Linforth, Ian D. Fisk

**Affiliations:** Division of Food Sciences, School of Biosciences, University of Nottingham, Sutton Bonington Campus, Loughborough, Leicestershire LE12 5RD, United Kingdom

**Keywords:** Chemometric techniques, Cheddar cheese, Cheese maturity, APCI-MS, PLS-DA, Volatile aroma compounds

## Abstract

Headspace techniques have been extensively employed in food analysis to measure volatile compounds, which play a central role in the perceived quality of food. In this study atmospheric pressure chemical ionisation-mass spectrometry (APCI-MS), coupled with gas chromatography–mass spectrometry (GC–MS), was used to investigate the complex mix of volatile compounds present in Cheddar cheeses of different maturity, processing and recipes to enable characterisation of the cheeses based on their ripening stages. Partial least squares-linear discriminant analysis (PLS-DA) provided a 70% success rate in correct prediction of the age of the cheeses based on their key headspace volatile profiles. In addition to predicting maturity, the analytical results coupled with chemometrics offered a rapid and detailed profiling of the volatile component of Cheddar cheeses, which could offer a new tool for quality assessment and accelerate product development.

## Highlights

• First application of APCI-MS with chemometrics for cheese classification.
• Volatile aroma compounds were correlated with cheese maturity.
• PLS-DA analysis gave 70% correct prediction of cheese maturity.

## 1 Introduction

The flavour of Cheddar cheese flavour is composed of a complex mixture of at least 180 compounds, including alcohols, aldehydes, ketones, esters, lactones, sulphides, free fatty acids and pyrazines (Curioni & Bosset, 2002), with diacetyl, (*Z*)-4-heptenal, methional, 2-acetyl-1-pyrroline, dimethyltrisulfide, 1-octen-3-one, (*Z*)-1,5-octadien-3-one, furaneol, (*Z*)-2-nonenal and (*E*)-2-nonenal, homofuraneol, acetic acid, and butyric acid identified as the most potent odorants (Milo & Reineccius, 1997; Zehentbauer & Reineccius, 2002). Throughout manufacture, cheese production represents a series of finely coordinated biochemical events, which, if synchronised and balanced, leads to products with highly desirable aromas and flavours, but if not balanced, off-tastes and off-aromas will result. Cheddar cheese requires an extended period of time to mature and develop the full flavour and texture of ripened cheese.

During maturation or ripening, microbiological and biochemical changes occur; bacteria and enzymes act on the fat, protein and carbohydrate in the cheese to produce the characteristic body, texture and flavour of the variety. The biochemical changes during ripening may be grouped into primary (proteolysis, lipolysis and metabolism of residual lactose, lactate and citrate) or secondary (metabolism of fatty acids and amino acids) events. It is these secondary reactions that result in the production of volatile flavour compounds that play a significant role in the flavour of cheese (McSweeney, Ottogalli, & Fox, 2004).

### 1.1 Proteolysis and lipolysis in Cheddar

Cheddar cheese ripening is mainly affected by the rate and extent of proteolysis. Proteolysis involves the hydrolysis of caseins to progressively smaller peptides and free amino acids by proteolytic enzymes such as rennet (Coker, 2003). Lactic acid bacteria (LAB) are also a major source of proteolytic enzymes in a wide variety of cheeses (De Wit, Osthoff, Viljoen, & Hugo, 2005). During cheese ripening, the enzymatic degradations of proteins and peptides lead ultimately to the formation of flavour-impact volatiles (Papetti & Carelli, 2013).

Cheese fat affects cheese flavour in several ways: firstly by acting as a source of aromatic compounds or precursors and secondly as a solvent for lipophilic flavour compounds produced from cheese lipids. Lipolysis results in the formation of free fatty acids (FFA), which are key constituents of Cheddar cheese flavour and can also be precursors of flavour compounds such as methyl ketones, alcohols and lactones (Smit, Vileg, Smit, Ayad, & Engels, 2002). Fatty acids are important and are often the predominant flavour components of many cheese types. During ripening, free fatty acids containing four or more carbon atoms may originate from milk fat lipolysis, due to the action of the endogenous milk lipases; lactic acid bacteria, present in starter cultures, are generally only weakly lipolytic. Short and moderate-chain, even numbered fatty acids significantly contribute to the characteristic note of Cheddar (Curioni & Bosset, 2002); the principal volatile fatty acids present during maturation include acetic, propanoic, butanoic, pentanoic, and hexanoic (De Wit et al., 2005). In general, much of cheese flavour is generated during lipolysis and proteolysis in addition to the catabolism of lactose and citrate by cheese micro flora.

Cheese maturation is a continuous process whereby cheese texture, flavour and aroma develop further over a period of time. For some cheese types the length of time of ripening is regulated by the development of certain attributes such as aroma and texture which are used to categorise the cheese prior to sale as mild, medium, mature, extra mature or vintage.

### 1.2 Chemometrics for dairy classification

There are manufacturing processes (e.g., addition of adjunct cultures) that can be applied to accelerate the rate of ripening. In these circumstances, methods for determining or predicting maturity are particularly useful (Coker, Crawford, Johnston, Singh, & Creamer, 2005). Reverse phase high-performance liquid chromatography (RP-HPLC) has been previously used to match cheeses to their maturity categories (Pham & Nakai, 1984). Various authors have also published studies on the classification of Cheddar cheese on the basis of maturity and quality. O’Shea, Uniacke-Lowe, and Fox (1996) used RP-HPLC to analyse the retentate and permeate of the water-soluble fractions (WSF) of 60 Cheddar cheese samples that varied in age and quality as determined by cheese graders but received poor differentiation of maturity. In addition there have been recent attempts using casein, peptide and amino acid profiles produced *via* electrophoretic and chromatographic methods to show correlations between sensory and instrumental textural information. Multivariate statistics have been applied to determine the influence of different manufacturing processes, types of raw milk and the incorporation of adventitious non-starter lactic acid bacteria strains on proteolysis and maturation (Ardo, Thage, & Madsen, 2002; Furtula, Nakai, Amantea, & Laleye, 1994; Skeie, Lindberg, & Narvhus, 2001), to provide information on the characteristics of the specific Cheddar cheese.

A rapid, simple, and reliable sample preparation method and FT-IR technique was developed for analysis of Cheddar cheese flavour quality. The infrared spectra could be correlated to specific flavour notes, such as fermented, sour, and unclean, and differentiated using multivariate classification models. This technique could then be used for the detection of flavour quality defects in Cheddar cheese and showed great promise as a rapid and simple tool for cheese analysis. Fourier-transform infrared microspectroscopy combined with multivariate analysis has also been employed to monitor the effect of adjunct cultures during Swiss cheese ripening.

Changes in sensory properties of Cheddar cheese during maturation were profiled by Muir, Hunter, Williams, and Brennan (1998) to give manufacturers an understanding of the quality of their individual cheeses. However there is no ‘best’ combination of analytical and statistical methods that can be used for every situation.

The aim of this research was to evaluate direct injection atmospheric pressure chemical ionisation-mass spectrometry (APCI-MS) coupled with gas chromatography–mass spectrometry (GC–MS) for its ability to identify and characterise the aroma volatiles of commercial Cheddar cheese manufactured in UK and predict the labelled age of Cheddar cheese using PLS-DA (partial least square-linear discriminant analysis) models. This will serve to complement sensory derived data sourced from trained panellists or experienced cheese graders.

## 2 Materials and methods

Five different commercial Cheddar cheese brands (coded V, W, X, Y, Z) comprising of five maturity grades: mild (MI), medium (ME), mature (M), extra mature (EM) and vintage (V) were ripened at 3–4 months, 10–12 months, 14 months and 18–36 months respectively. Cheeses were grated and about a gram of cheese alone was placed into glass bottles for headspace analysis. A total of 52 Cheddar cheese samples (with triplicates) was prepared and stored at 4 °C until analysed; samples were selected to cover the breadth of the UK Cheddar market and included major UK Cheddar brands.

### 2.1 GC–MS analysis

Headspace solid phase microextraction (HS-SPME) coupled to gas chromatography–mass spectrometry (GC–MS) was applied to analyse the volatile compounds of Cheddar cheese samples. An automated SPME sampling unit (CombiPal. Zwingen, Switzerland) was used with a 2-cm length StableFlex SPME fibre with 50/30 μm divinylbenzene/Carboxen on polydimethylsiloxane coating (DVB/CAR/PDMS), purchased from Supelco (Bellefonte, PA). This was exposed to the headspace for 30 min. The cheese samples were stirred for 30 min at 60 °C to accelerate the equilibrium of headspace volatile compounds between the cheese matrix and the headspace. Analysis of the volatiles was performed on a Trace GC Ultra (Thermo Scientific, Waltham, MA) that was attached to an ISQ series mass spectrometer (Thermo Scientific), carried out in electron ionisation mode with an ion source temperature of 200 °C, and a scanned mass range of *m*/*z* 15–200. The gas chromatograph was equipped with a ZB-Wax fused silica capillary column (100% polyethylene glycol phase, 30 m × 0.25 mm × 1.0 μm; Phenomenex, Torrance, CA). The GC oven was held at 40 °C for 2 min then heated to 250 °C at 4 °C/min. The GC to MS transfer line was maintained at 250 °C. Helium was the carrier gas with a constant flow rate of 1.0 mL/min in splitless mode. Cheddar cheeses were analysed in triplicate. Aroma compounds were identified by comparison of their retention times with authentic standards and validated against a mass spectral library (NIST/EPA/NIH).

### 2.2 APCI-MS

The cheese samples were analysed in full scan mode, using the MS Nose interface (Micromass, Manchester, UK) fitted to a Quattro Ultima mass spectrometer (Waters Corporation, Milford, MA) (Fernández-Vázquez et al., 2013). Following the method described by Gan, Soukoulis, and Fisk (2014), ions of mass to charge (*m/z*) ratios from 40–200 were monitored. The intensity of these ions was measured at a cone voltage of 20 V, source temperature of 75 °C and dwell time of 0.5 s. All analyses were run in triplicate (Ashraf, Linforth, Bealin-Kelly, Smart, & Taylor, 2010).

### 2.3 Statistical analysis

Data from MS Nose were exported using Waters Masslynx™ Software version 4.1, whereas data from GC–MS were processed with Thermo Scientific™ Xcalibur™ Software prior to chemometric analysis using The Unscrambler software (version 9.7, CAMO AS, Norway). The chemometric approach composed of principal component analysis (PCA) and partial least squares regression (PLS) performed with full cross validation to classify the maturity of the cheese. Full cross validation is an evaluation tool to check calibration models, based on systematically removing samples in the model and testing the performance of the model using the remaining data set. The maximum number of factors in both PCA and PLS models were selected by the criterion of the lowest number of factors that gave the closest to minimum value of predicted residual error sum of squares function, in order to avoid over fitting of the data (Cozzolino et al., 2008). All variables were weighted (1/standard deviation) prior to chemometrics application, so that drifts and baseline effects were removed. PLS regression was used to determine the relationship between multiple dependent predictor variables (GC and APCI data) and the maturity/age of cheese. Furthermore it was used as an exploratory analysis tool to select suitable predictor variables for predictive linear modelling. PLS was applied to model the maturity/ age of cheese using those aroma volatiles with predictive ability.

## 3 Results and discussion

### 3.1 Headspace analysis by GC–MS and APCI-MS

Twenty-three volatile aroma compounds were detected and identified in the headspace of the grated Cheddar cheeses (Table 1) by SPME-GC–MS. These compounds were mainly aldehydes, ketones and carboxylic acids, which were found to be similar to previously published data for the characteristic flavour profile of aged cheeses (Biasioli et al., 2006; Coker et al., 2005; Milo & Reineccius, 1997; Whetstine, Drake, Nelson, & Barbano, 2006; Zehentbauer & Reineccius, 2002).

**Fig. 1:**
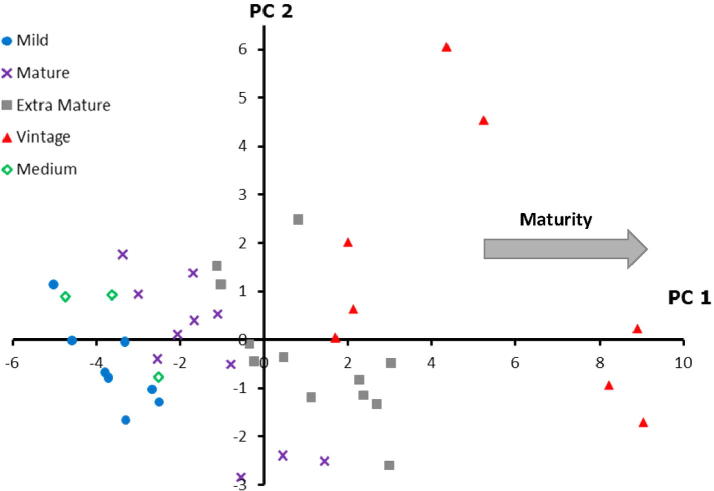
Principal components analysis (PCA) on the static headspace data (averaged, mean centred and auto-scaled) obtained by GC–MS and APCI-MS analysis of the 42 grated Cheddar cheeses (‘calibration’ set). PC1 and PC2 account for 28% and 7% of the variance respectively.

Principal component analysis (PCA) was performed to evaluate the variance associated with the samples as described by the GC–MS and APCI-MS data. Forty-two calibration Cheddar cheese samples and a total of 47 key volatiles (both GC–MS and APCI-MS) that exhibited significance in classifying Cheddar maturity were used. Interpretation of the dimensions of the PCA was facilitated by inspection of the vector loadings and the correlation coefficients of the scores of the cheese samples to avoid overfitting. As shown in Fig. 1, the first principal component (PC1) accounted for 28% of the aroma compounds and was mainly associated with maturity. PC1 and PC2 accounted for 35% of total variance of the spectral data. The lowest residual variance was found with 7 PCs, however the model using 2 PCs appeared sufficiently robust and explicit, containing variables from GC–MS and APCI-MS to illustrate the maturity of the cheeses.

**Fig. 2:**
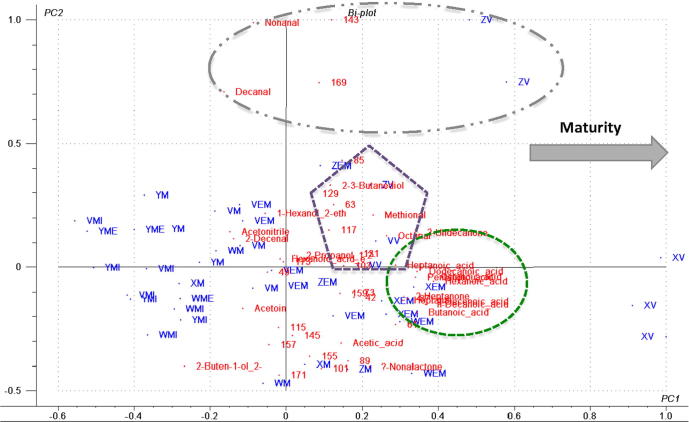
Principal components analysis (PCA) biplot on the different types of ‘calibration’ cheeses and static headspace obtained by GC–MS and APCI-MS analysis of the grated Cheddar cheeses. Samples were randomly grouped into ‘calibration’ and ‘prediction’ sets. The different brands were indicated by the different prefixes: ‘V’, ‘W’, ‘X’, ‘Y’ and ‘Z’, whereas the different maturity of cheese was marked by the different suffixes: -MI for mild, -ME for medium, -M for mature, -EM for extra mature and -V for vintage. (Maturity grading was based on labelling printed on packaging).

As the Cheddar matures, more aroma volatiles, especially acids (such as butanoic, pentanoic, hexanoic acids) and ketones, like 2-heptanone, 2-undecanone, were produced (indicated by the smaller oval in Fig. 2). The more mature cheeses also displayed an apparent cheesier, meatier and more fatty profile with higher concentrations of characteristic aromas, such as 2,3-butanediol, octanal and methional (a common sulphur-compound in cheese marked by the pentagon in Fig. 2). This corresponds to previously published work identifying the potent aroma compounds in Cheddar cheese, in which methional was shown to be a potent aroma compound. The apparent lack of other potent aroma compounds previously identified is of interest and probably illustrates the variation in abundance in natural products, this is further supported by the disparity in potent aroma compounds identified by different groups on different Cheddar cheeses (Milo & Reineccius, 1997; Zehentbauer & Reineccius, 2002); interestingly both papers herein cited found methional as a potent aroma compound in addition to a number of other compounds, most of which were different between the papers.

The biplot shown in Fig. 2 provides an insight into the role played by the raw APCI-MS data. It shows a positive relationship for the ester peaks (*m*/*z *= 117, *m*/*z* = 131, *m*/*z* = 85, *m*/*z* = 103) with the vintage series (V) (marked by the pentagon in Fig. 2). Even though APCI-MS could not provide unambiguous identification of aroma compounds, literature data and comparison with other data enabled the tentative identification of many peaks. The protonated masses *m/z *= 89 (butanoic acid), *m*/*z *= 101 (2-hexanone), *m*/*z *= 155 (decanoic acid) and *m*/*z *= 157 (2-decanone) appeared to be important volatiles that contribute to cheese profiles (Biasioli et al., 2006). Overall ripening resulted in a progressive increase in aroma in the headspace of the Cheddar cheeses.

**Fig. 3:**
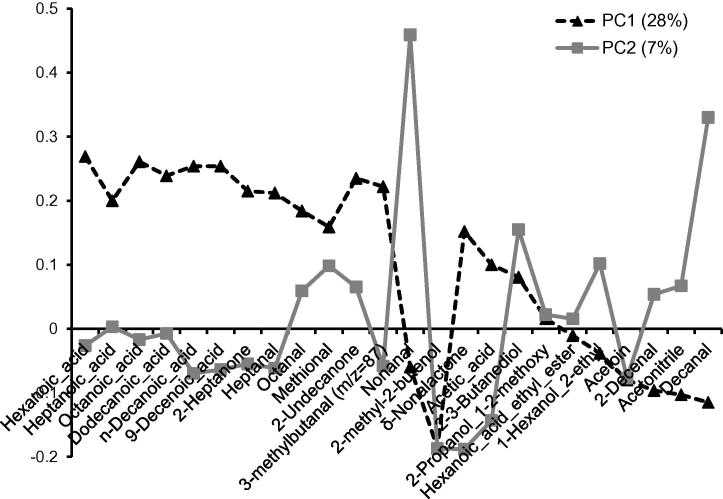
PLS-DA loadings for the first two factors of the classification models based on both the GC–MS and APCI-MS data obtained by the static headspace analysis of the Cheddar cheeses.

Alignment to PC1 and PC2 provided evidence of the relative importance of each of the aroma volatile to the groups of Cheddar cheese samples. Increasing levels of butanoic, pentanoic, hexanoic, heptanoic, octanoic, decanoic, 9-decenoic, dodecanoic acids, 2-heptanone, heptanal, octanal, methional, 2-undecanone and 3-methylbutanal (*m*/*z *= 87) were correlated with PC1; whereas nonanal and 2-methyl-2-buten-1-ol were aligned with PC2 (Fig. 3). Whilst there were some brand-specific differences (ZV cheeses were rich in nonanal), there was a general consensus across the brands and a strong correlation of PC1 with maturity. Whetstine et al. (2006) had also identified these acids as predominant odorants in aged Cheddar, Christensen and Reineccius (1995) identified 3-methyl-butanal, ethyl butyrate (*m/z *= 117) and 1-octen-3-ol (*m/z *= 129) as 3 of the 25 potent odorants in aged Cheddar; these aromas were seen to have strong relationships with the vintage Cheddar group. 2-Methyl-2-buten-1-ol and δ-nonalactone on PC2 were also significant, but more associated with the mature (M) categories of cheese.

Acetoin is the major volatile compound in the headspace of fresh milk which was seen to decrease with ripening, presumably due to its reduction to 2-propanol and 2,3-butanediol, as a result of fermentation by adventitious bacteria (Dimos, Urbach, & Miller, 1996). Keen, Walker, & Pederby, 1974 also showed that diacetyl (2,3-butanedione), with a protonated mass of 87, is a natural by-product of fermentation, which was dominant in the more mature Cheddar cheeses, especially the Extra Mature ones (marked by small oval) on the right hand side of the PCA biplot (Fig. 2).

Acetic acid is the major free alkanoic acid in Cheddar. A steady increase in the level of acetic acid over the whole period of maturation was discovered previously by two groups (Dimos et al., 1996; Urbach, 1993). This was depicted in Fig. 2 where acetic acid was correlated to Cheddar of mature grade. Diacetyl formed from the dehydration of 2,3-butanediol, is a common volatile present in dairy products and showed a close association with the mild Cheddar cheese; 2,3-butanediol had a positive correlation with the more matured Cheddar ‘ZV’ (Fig. 2).

### 3.2 Prediction of Cheddar cheese maturity

GC–MS and APCI-MS were able to successfully build a predictive model for maturity across the broad range of samples used within the model. Hence, PLS regression models were built to interpret and predict the age of Cheddar cheese using headspace data from GC–MS and APCI-MS. PLS combines the properties of multiple linear regression and PCA to make linear combinations in the dependent matrix. By means of these regression models, the relationships between aroma compounds and the age/maturity of Cheddar cheese were established. Parameters used to evaluate the models prediction ability were: root mean square error prediction (RMSEP) and coefficient of determination (*r*^2^) for the derived model between actual and predicted *Y*-variables (Capone et al., 2013). RMSEP indicates the absolute fit of the model to the data and is a good measure of how accurately the model predicts the response. The RMSEP and *r*^2^ values for the prediction model were 3.94 and 0.85 respectively.

**Fig. 4:**
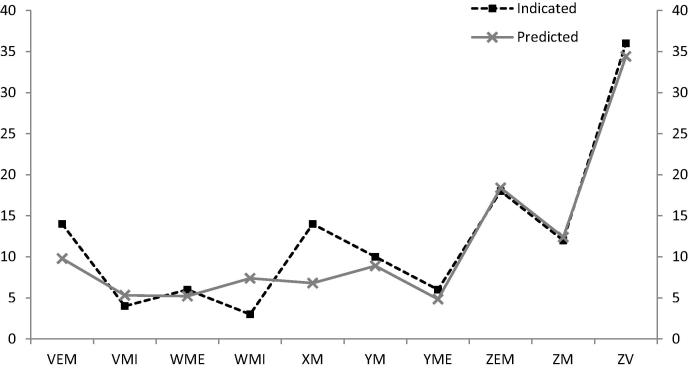
Comparison of the age of 10 Cheddar cheeses (‘predicted’ set) given by the manufacturer (labelled as *‘indicated’*) and predictive values from regression model (labelled as *‘predicted’*).

2-methyl-2-buten-1-ol, hexanoic acid, heptanoic acid, octanoic acid, 9-decenoic acid, acetoin and 2-decenal were statistically significant in predicting the age/maturity of Cheddar cheese in the predictive model. The development of acids in extra mature and vintage cheeses seems to be the main driver that differentiates the classified age of the cheese. Furthermore, 2-decenal and acetoin distinguished the mature cheeses from the mild and medium ones (Fig. 2). Lipolysis, during maturation, results in the formation of free fatty acids (FFA), which are key constituents of Cheddar cheese flavour. The estimates of Cheddar cheese age supplied by the manufacturers, compared to those predicted by the PLS model for the 10 prediction samples used, are shown in Fig. 4, where the age of the Cheddar was 70% correctly predicted. For the validation of the method for cheese graders and its potential use as a rapid screening tool within new product development, the predictive power of the herein constructed model was satisfactory, but we recommend further validation prior to commercial use.

**Fig. 5:**
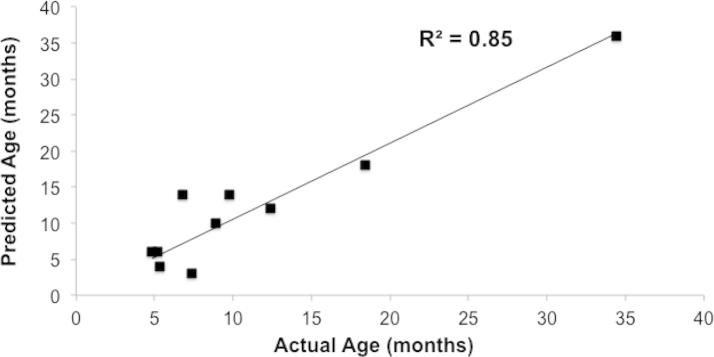
Predictive age of Cheddar cheeses from model (labelled as *‘Predicted’*) against age based on actual storage (labelled as *‘Actual’*).

It is well known that there are several environmental factors in addition to processing strategies and production practices, which contribute to the final free fatty acids (FFA) content within cheeses. These include the milk quality, seasonal variation, heat treatment, lactic acid starters used, ripening and storage temperature, brine concentration and enzymes found in rennet. It is therefore very promising that despite not knowing the manufacturing conditions for the cheeses, a model could be developed with a prediction coefficient of determination (*r*^2^) of 0.85 for predictive age against actual storage (Fig. 5).

## 4 Conclusion

GC–MS and APCI-MS headspace analysis were fast (200–500 samples per day) and effective techniques for determining aroma compounds relevant to Cheddar cheese. Characterisation of Cheddar cheese maturity based on headspace measurements using GC–MS and/ or APCI-MS combined with chemometric treatment of data was shown to be effective when presented within a PCA format. With the PLS-DA chemometric approach, it was possible to classify and predict the age of the Cheddar cheeses on the basis of their headspace aroma. The PLS model generated was robust enough to accurately predict 70% of the Cheddar cheeses using the aroma compounds from the headspace data alone. This further established the applicability of this multivariate statistical technique as a tool to monitor the quality of foodstuffs.

## Figures and Tables

**Table 1:** Volatile compounds (MW, molecular weight) identified in the headspace above grated Cheddar cheese using SPME-GC–MS.

Aroma volatile (MW)	Retention time (min)	Description^1^
Acetonitrile (41)^a^	3.40	Solvent-like
1-Ethoxy-2-propanol (104)^a^	6.59	Solvent-like
2-Heptanone (114)^a^	7.06	Banana; cinnamon; spicy
Heptanal (114)^a^	7.12	Fatty; oily
Diacetyl (86)^a^	7.13	Buttery flavour
Acetoin (88)^a^	10.03	Butter; creamy
Octanal (128)^a^	10.56	Fatty; citrus
2-Methyl-2-buten-1-ol (86)	11.31	Green; fruity
Ethyl-octanoate (172)^a^	14.92	Apricot; banana; floral; pear; wine-like
Methional (104)^a^	15.44	Beef; cheese; creamy; meaty; oily
Acetic acid (60)	16.45	Vinegar
2-Ethyl-1-hexanol (130)^a^	16.71	Oily; rose
2-Undecanone (170)^a^	19.90	Citrus; fruity; rose
2-Decenal (154)^a^	21.15	Fatty; green; meaty; oily
Butyric acid (88)^a^	21.67	Rancid cheese
Pentanoic acid (102)^a^	24.85	Fatty; earthy
Hexanoic acid (116)^a^	27.82	Cheese; fatty; sour
δ-Nonalactone (156)^a^	29.88	Butter; meaty; nutty; sweet
Heptanoic acid (130)^a^	30.66	Cheese
Octanoic acid (144)^a^	33.35	Cheese; oily
Decanoic acid (172)^a^	38.45	Fatty; citrus
9-Decenoic acid (170)^a^	39.80	Soapy
Dodecanoic acid (200)^a^	43.08	Fatty

1 Whetstine et al. (2006).

a Significant difference between maturity stages ANOVA-Tukey (*p* < 0.05).
